# miR-193b-5p and miR-374b-5p Are Aberrantly Expressed in Endometriosis and Suppress Endometrial Cell Migration In Vitro

**DOI:** 10.3390/biom14111400

**Published:** 2024-11-03

**Authors:** Caroline Frisendahl, Yiqun Tang, Nageswara Rao Boggavarapu, Maire Peters, Parameswaran Grace Lalitkumar, Terhi T. Piltonen, Riikka K. Arffman, Andres Salumets, Martin Götte, Eberhard Korsching, Kristina Gemzell-Danielsson

**Affiliations:** 1 WHO Collaborating Centre, Division of Neonatology, Obstetrics, Gynecology, and Reproductive Health, Department of Women’s and Children’s Health, Karolinska University Hospital, Karolinska Institutet, SE 17176 Stockholm, Sweden; caroline.frisendahl@ki.se (C.F.); yiqun.tang@ki.se (Y.T.); nageswara.boggavarapu@ki.se (N.R.B.); lalit.kumar@ki.se (P.G.L.); 2 Department of Obstetrics and Gynecology, Research Unit of Clinical Medicine, Medical Research Centre, Oulu University Hospital, University of Oulu, 90220 Oulu, Finland; terhi.piltonen@oulu.fi (T.T.P.); riikka.arffman@oulu.fi (R.K.A.); 3 Department of Obstetrics and Gynecology, Institute of Clinical Medicine, University of Tartu, 50406 Tartu, Estonia; maire.peters@ut.ee (M.P.); andres.salumets@ki.se (A.S.); 4 Celvia CC, Competence Centre on Health Technologies, 50411 Tartu, Estonia; 5 Division of Obstetrics and Gynecology, Department of Clinical Science, Intervention and Technology (CLINTEC), Karolinska Institutet, and Karolinska University Hospital, SE 17177 Stockholm, Sweden; 6 Department of Gynecology and Obstetrics, University Hospital of Münster, University of Münster, 48149 Münster, Germany; martin.goette@ukmuenster.de; 7 Institute of Bioinformatics, University Hospital of Münster, University of Münster, 48149 Münster, Germany; eberhard.korsching@uni-muenster.de

**Keywords:** endometriosis, endometrium, endometrioma, small RNA sequencing, mRNA sequencing, microRNAs, cell migration, cell proliferation, bioinformatic analysis

## Abstract

(1) Background: Endometriosis is a highly prevalent gynecological disease affecting 10% of women of reproductive age worldwide. miRNAs may play a role in endometriosis, though their exact function remains unclear. This study aimed to identify differentially expressed miRNAs in endometriosis and study their functions in the disease. (2) Methods: Endometrial tissue was collected from women with endometriosis (n = 15) and non-endometriosis controls (n = 17). Dysregulated miRNAs were identified through small RNA-sequencing, and their biological significance was explored by target gene prediction and pathway analysis. Selected miRNAs were examined in paired ectopic endometriomas and eutopic endometrium (n = 10) using qRT-PCR. Their roles in cell migration and proliferation were further examined in vitro using functional assays. To identify potential target genes, we performed mRNA sequencing on transfected cells and the endometrioma cohort. (3) Results: We identified 14 dysregulated miRNAs in the eutopic endometrium of women with endometriosis compared to endometrial tissue from women without endometriosis. Pathway analysis indicated enrichment in cell migration and proliferation-associated pathways. Further ex vivo studies of miR-193b-5p and miR-374b-5p showed that both miRNAs were upregulated in endometrioma. Overexpression of these two miRNAs in vitro inhibited cell migration, and mRNA sequencing revealed several migration-related genes that are targeted by these miRNAs. (4) Conclusions: Our study identified two key endometrial miRNAs that may be involved in the pathogenesis of endometriosis by regulating cell migration.

## 1. Introduction

Endometriosis is a highly prevalent chronic disease affecting approximately 10% of reproductive-age women, and is characterized by the presence of endometrial epithelial and stromal cells outside of the uterine cavity [1]. It can develop from an early age and usually takes more than 6 years from the onset of symptoms to receive a diagnosis [2]. At present, there are no definitive curative solutions, largely due to the unclear etiology and pathogenesis. Ultrasound plays a role in diagnosing endometriosis; however, the invasive laparoscopy combined with histological examination of ectopic lesions is still the current standard method to diagnose the disease, due to a lack of precise nonsurgical options [3].

MicroRNAs (miRNAs) are small non-coding RNAs of 20–22 nucleotides in length that act as post-transcriptional gene repressors, interacting with up to hundreds of target mRNAs [4,5,6]. Through various post-transcriptional regulatory mechanisms, dysregulated miRNAs have been found to either up- or downregulate biological and cellular processes such as migration, proliferation, invasion, apoptosis, and stem cell maintenance [7]. Many researchers believe that aberrant miRNA expression may contribute to the development and progression of endometriosis [8,9,10,11]. For instance, it has been reported that downregulation of miRNA-126-5p can induce *BCAR3* expression, which promotes cell migration and invasion in endometriosis [12]. Although abnormal miRNA profiles in eutopic and ectopic endometria from women with endometriosis have been reported in many studies, the direction of miRNA gene expression has been inconsistent across studies [13]. This could be related to differences in experimental design, lack of proper biological and functional validations, and differences in clinical characteristics of the included study populations [13]. Additionally, few studies have compared eutopic endometrial tissues from women with endometriosis and healthy controls in the proliferative phase of the menstrual cycle from women with stage III–IV endometriosis.

Therefore, this study aimed to conduct an unbiased screening to identify differentially expressed miRNAs in the proliferative eutopic endometrium of patients with stage III–IV endometriosis and in women without diagnosed endometriosis. Further, we aimed to validate the expression profile of selected miRNAs and their functional role in the pathogenesis of endometriosis. We hope to provide novel insights into the molecular pathogenesis of endometriosis.

## 2. Materials and Methods

### 2.1. Study Population and Sample Collection

All samples were collected at Tartu University Hospital, Estonia from 2010 to 2018. In the first cohort, 15 women with laparoscopically confirmed endometriosis (stage III–IV) and 17 age- and BMI-matched controls who were laparoscopically confirmed to be free of endometriosis were included. Exclusion criteria included infections, endocrine or metabolic disorders, anatomic abnormalities, autoimmune diseases, and carcinomas. All control women had regular menstrual cycles and proven fertility (at least one live birth). In the second cohort, paired ectopic ovarian endometrioma tissue and eutopic endometrial tissue were collected from 10 patients with endometrioma (Stage III–IV), with the same exclusion criteria as applied for the first cohort. All women included in the study had been free of any hormonal medication for a minimum of 3 months before surgery. The diagnosis of endometriosis was confirmed by a pathologist and classified according to the ASRM guideline [14]. All endometrial biopsies were collected according to a previously published protocol [15].

### 2.2. Ethics

The study was approved by the Research Ethics Committee of the University of Tartu (337/T-5 and 333/T-6) and Karolinska Institutet (Original permit (Dnr 2016/95-31/4) and Amendments (Dnr 2022-04929-02 and Dnr 2024-03617-02)). All patients gave written informed consent before inclusion in this study.

### 2.3. Cell Lines

The human immortalized epithelial endometriotic cell line (12Z) had been donated as a kind gift by Anna Starzinski-Powitz and the endometrial stromal cell line (HESC) (cat. No T0533) was purchased from ABM (Richmond, BC, Canada). The 12Z cells were cultured in Dulbecco’s modified Eagle’s medium (DMEM, Gibco, Cat. 41965-039, Thermo Fisher Scientific, Inc., Waltham, MA, USA) with 10% fetal bovine serum (FBS) (Gibco, Thermo Fisher Scientific, Inc., Waltham, MA, USA) and 1% penicillin/streptomycin (Gibco, Cat. 15140-122, Thermo Fisher Scientific, Inc.) at 37 °C with 5% CO_2_. HESC cells were cultured in DMEM containing 10% FBS, 1% penicillin-streptomycin, and 250 μL insulin (10 mg/mL) (Sigma, WI, USA, Cat. I0516) at 37 °C with 5% CO_2_.

### 2.4. Total RNA Extraction

Total RNA was isolated from endometrial and endometriotic tissues and cell lines using the Zymo quick RNA-microprep kit (Zymo Research, CA, USA, Cat. R1501) according to the manufacturer’s instructions. Briefly, the tissues and RNA lysis buffer were added to a cryotube with Zymo lysis beads and mixed in a tissue disrupter for 15 min, whereas the cells were lysed directly by mixing the lysis buffer with the cells, followed by 2 min of incubation. The endometrioma samples were disrupted using a tissue disrupter for one minute. After centrifugation, the supernatant was transferred to a Zymo-spin IC column, followed by DNAse treatment and several washing steps in ethanol. Extracted RNA was resuspended in 20 μL of RNAse-free water. RNA concentration (ng/μL) was measured by Qubit™ Flex Fluorometer (Invitrogen, CA, USA, Cat. Q33327) and stored at −80 °C.

### 2.5. Small RNA Library Preparation and Sequencing

Small RNA cDNA libraries were constructed for all endometrial samples in the first cohort, according to a previously published protocol [16]. A total of 1 ng of RNA per sample was used as input concentration. The amplified libraries were purified with AMPure XP beads for the samples in a 1:1 ratio. Quantification of the amplified libraries was performed using the Qubit Flex Fluorometer (Invitrogen, CA, USA, Cat. Q33327) and the Qubit 1X dsDNA high-sensitivity kit (Invitrogen, CA, USA, Cat. Q33230). The Agilent 2100 Bioanalyzer system (Agilent, Santa Clara, CA, USA) and the Agilent high-sensitivity DNA kit (Agilent, Santa Clara, CA, USA Cat. 5067-4627) were used to check the quality of the cDNA libraries. A total of 10 ng of each library was pooled and sequenced in the Illumina NextSeq 550 platform with 1 × 75 base pairs, and single end reads, at the Bioinformatics and Expression Analysis core facility at Karolinska University Hospital, Sweden.

### 2.6. mRNA Library Preparation and Sequencing

cDNA libraries for next-generation mRNA sequencing were constructed for the mimic transfected 12Z and HESC cell lines (n = 3 for each condition) and the paired endometrioma and endometrial biopsies included in cohort 2, using the Smart-seq2 protocol [17] with 1 ng of total RNA as input concentration for each sample. The enzymatic fragmentation and tagmentation of cDNA were performed using the Nextera XT kit (Illumina Inc., San Diego, CA, USA), along with IDT^®^ for Illumina^®^ DNA Unique Dual Index barcodes. The final amplified libraries were purified with AMPure XP beads at a ratio of 1:1 (sample versus beads). The final cDNA library was quantified using a Qubit 1X HS DNA assay kit (Thermo Fisher Scientific, Waltham, MA, USA, Invitrogen), and the quality of the libraries was analyzed using a 2100 Bioanalyzer system (Agilent, Santa Clara, CA, USA) with a high-sensitivity DNA chip (Agilent, Santa Clara, CA, USA). An equimolar concentration of 5 ng from each library was pooled and sequenced on an Illumina NovaSeq 6000 sequencing platform (Novogene, Cambridge, UK) with a 2 × 150 bp read setup.

### 2.7. Sequencing Data Processing and Bioinformatic Analysis

Small RNA sequencing data analysis: The small RNA bioinformatic analyses were performed according to a previously published pipeline, with minor modifications [18]. The raw FASTQ files were quality-checked using FastQC (Version 0.11.9). The UMIs were removed and appended to the read header for later analysis, followed by trimming of the Illumina 3′ adapters and the CA bases linked to the UMI. The trimmed reads were aligned to the human genome 38 (hg38) using STAR (version 2.7.2a). Aligned reads were filtered for a read length of 40nt, followed by UMI deduplication and removal of precursor molecules. Quantified and aligned reads were annotated for miRNAs using Mirbase (version 22). Differential expression analysis was performed using the DESeq2 tool (version 1.24.0). Differentially expressed genes with an FDR < 0.05 and fold change of <−2 or >˃2 were considered significant.

mRNA sequencing data analysis: the raw mRNA sequencing data processing and analysis were performed using the Partek^®^ Flow^®^ platform (version 10.0.22.1005). A raw-data quality check was conducted using the FastQC (version 0.11.9) toolkit in the Partek Flow platform. Briefly, after trimming the Nextera adapters, the trimmed reads were aligned to hg38 using a STAR aligner toolkit. An alignment rate of 80% was used as a cut-off value for each sample. After alignment, mitochondrial and ribosomal genes, as well as genes with a count of <10 in at least 80% of the samples, were filtered out. The data were normalized using the median ratio method to avoid variations in the read counts in the samples. For the downstream analysis, we split the data into two groups that were analyzed separately: one is the miRNA-transfected cell lines, and the other group is the endometrioma cohort. For the cell line data, we separated the samples into four comparison groups for further analysis: (1) miR-193b-5p transfected 12Z cells and mimic controls, (2) miR-374b-5p transfected 12Z cells and mimic controls, (3) miR-193b-5p transfected HESC cells and mimic controls and (4) miR-374b-5p transfected HESC cells and mimic controls. A differential expression analysis of the filtered counts was performed using the DESeq2 tool (version 3.5). Genes with low expression levels in all samples, e.g., fewer than 10 counts, were excluded. We applied a false discovery rate (FDR) of <0.05 and fold change (FC) of <−1.5 or ˃1.5, since the cell lines are relatively homogenous. The fold-change cut-off value for the endometrioma cohort was, however, set as 2. The KEGG (Kyoto Encyclopedia of Genes and Genomes) database was used to carry out a pathway enrichment analysis of differentially expressed genes (DEGs) [19]. A *p*-value < 0.05 was considered significant for enriched biological signaling pathways.

### 2.8. Target Gene Prediction and Pathway Analysis

Target genes of the differentially expressed miRNAs were predicted using a web-based tool miRTarBase [20]. Biological KEGG pathways were further predicted separately for the miRNA target genes and the differentially expressed genes in the mRNA sequencing data, using the web-based tool g: Profiler (version e111_eg58_p18_30541362), by utilizing the default g:SCS algorithm. An adjusted *p*-value of <0.05 was considered statistically significant.

### 2.9. Transfection of miRNA Mimics

The 12Z and HESC cells were cultured in 6-well plates (Sarstedt, Cat. 83.3920, Numbrecht, Germany) and transfected with hsa-miR-193b-5p mimics, hsa-miR-374b-5p mimics, miRNA mimic positive control (PC) and miRNA mimic negative control (NC) miRVana (Ambion by life technologies, Austin, TX, USA). Transfection was conducted using Dharmacon Dharmafect (Cat. T-2001-03). After 24 h of incubation, the transfection media was changed to normal media. The transfection efficiency was confirmed using qRT-PCR, by checking the successful downregulation of the target gene (TWF1) after transfection with the positive control miRNA (miR-1), as well as the successful upregulation of the transfected miRNAs. The cells were harvested 48 h after transfection for migration and proliferation assays and 72 h after transfection for qRT-PCR analysis.

### 2.10. qRT-PCR

Selected differentially expressed miRNAs identified by small RNA sequencing were examined in the endometrioma cohort using qRT-PCR. Reverse transcription from RNA to cDNA was performed using the SuperScript VILO^TM^ kit (Invitrogen, CA, USA, Cat.11754050) and 18S was used as a reference gene for mRNA genes. Meanwhile, cDNA was synthesized for miRNAs using the TaqMan™ MicroRNA Reverse Transcription Kit (Applied Biosystems, Cat.4366596) and the 5× TaqMan microRNA assays on a BIOER Life Touch thermal cycler (Techtum, Sweden). qRT-PCR was performed using the corresponding 20× TaqMan microRNA assay and TaqMan™ Fast Advanced Master Mix (Applied Biosystems, CA, USA, Cat.4444557) on a One Step Plus Real-time PCR system (Applied Biosystems, Foster City, CA, USA), according to the manufacturer’s protocol. Each sample was run in triplicate, and hsa-miR-23b-3p, showing a stable expression in endometrial tissue, was used as a housekeeping gene for the miRNAs. Transfection efficiency was confirmed by qRT-PCR. Data were analyzed using the standard 2^−ΔΔCT^ method.

### 2.11. Wound Healing Assay

The horizontal migration ability of 12Z and HESC was assessed by using a wound healing assay. After 48 h of transfection, cells cultured in 6-well plates were seeded into culture-insert 2-well dishes (ibidi, Cat. 81176, Munchen, Germany) at a cell density of 300,000 cells/mL, cultured overnight. Wounds were created by removing the insert when the cells reached 100% confluency. Debris and non-adherent cells were eliminated by rinsing with 1 mL DPBS (Gibco, Carlsbad, CA, USA), and DMEM (Gibco, Carlsbad, CA, USA) with 1% serum were then added to the culture dish. Photos were captured by a light microscope at different time points after the gap was created (0 h, 6 h, 12 h, 18 h, 24 h for 12Z and 0 h, 12 h, 24 h, 36 h for HESC) to record the cell-free area. The cell-free area at each time point was captured in three fields per culture dish under 10× magnification. The percentage of area covered by the cells at each time point was calculated and analyzed by Fiji (v2.16.0). The cell-free area at each time point was subtracted from the 0 h cell-free area and results were expressed as the percentage of the covered area. Each condition and time point was performed in triplicate.

### 2.12. Transwell Cell Migration Assay

The vertical migration ability of mimic transfected 12Z and HESC cells was assessed using a transwell migration assay in a 24-well culture plate (Cat. 353504, Corning, NY, USA) and Falcon^®^ Permeable Support for 24-well Plate with 8.0 µm Transparent PET Membrane (Cell culture inserts, Falcon, Corning, Cat. 353097). The miRNA mimic and mimic-control transfected cells were digested and suspended at a density of 20,000 cells/mL using a serum-free culture medium. The cell suspension was then added to the top chamber (100 μL per chamber). The bottom chamber was supplemented with culture media with 10% fetal bovine serum (FBS) as the chemoattractant. After 12 h of incubation at 37 °C, the cells that had migrated through the membrane were fixed with methanol for 4 min, washed with PBS, and finally stained with 1% crystal violet for 4 min. After washing the chamber several times in distilled water, the cells staying on the upper membrane were removed with a cotton swab. Migrated cells were counted in 4 random fields under a 5× objective lens and imaged using a light microscope (Nikon, Tokyo, Japan). Fiji (v2.16.0) was used for cell counting. Three independent experiments were conducted for each condition.

### 2.13. BrdU cell Proliferation Assay

To investigate the proliferative ability of cells after transfection with miRNA mimics in vitro, a BrdU incorporation assay was conducted using the BrdU cell proliferation ELISA kit (colorimetric) (Cat. 126556, Abcam, Cambridge, UK). Briefly, 12Z and HESC cells were cultured and seeded in flat-bottomed 96-well plates at a density of 1000 cells/100 μL/well in complete culture media. A total of 20 μL 1 × BrdU reagent was added to each well to label the cells. After 20 h of incubation at 37 °C with 5% CO_2_, the culture medium was removed, and a fixing solution was added. Primary anti-BrdU monoclonal detected antibody (100 μL/well) was added to each well for 1 h at room temperature. Subsequently, a peroxidase Goat Anti Mouse IgG Conjugate was added for 30 min incubation at room temperature. After removing the antibody conjugate, the cells were washed, supplemented with 100 μL TMB peroxidase substrate per well, and incubated in the dark for 30 min. A fluorescence microplate reader (Fluostar^®^ Omega, Berlin, Germany, BMG) was used to measure the absorbance at 450 nm, following the manufacturer’s instructions. The value of fluorescence intensity is proportional to the amount of BrdU incorporation in the proliferating cells. Each experiment was performed in triplicate.

### 2.14. Statistical Analysis

All statistical analyses in our study were performed by GraphPad Prisma 9.0 software. Data are presented as the mean ± SD. Demographic data were compared between the endometriosis and control groups using the non-parametric Mann–Whitney U-test. BrdU, transwell, and wound healing assay data were calculated by the non-parametric Mann–Whitney U-test. Differences in gene expression of miRNAs in the paired endometrioma and endometrium cohort were calculated using the Wilcoxon matched-pairs signed-rank test. A *p*-value < 0.05 was considered statistically significant.

## 3. Results

### 3.1. DE-miRNAs in Eutopic Endometrium from Women with and Without Endometriosis

**Figure 1:**
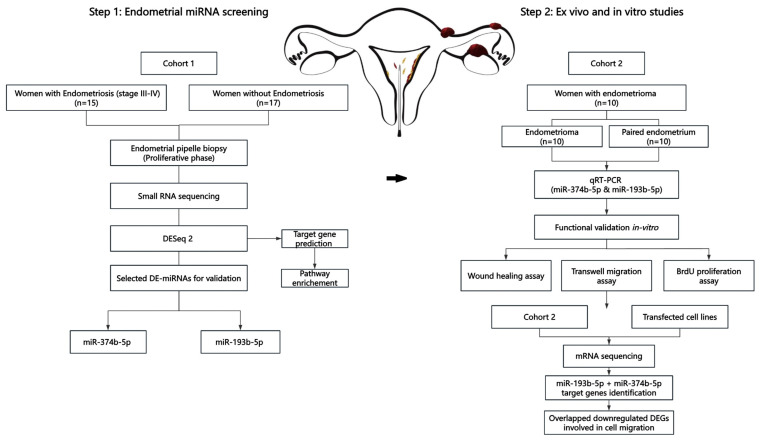
Overall study design flowchart. DE-miRNAs: differentially expressed miRNAs; DEGs: differentially expressed genes.

As shown in the study flowchart (Figure 1), small RNA sequencing was conducted to identify differentially expressed miRNAs in eutopic endometrium between women with endometriosis (n = 15) and without endometriosis (controls n = 17). The clinical characteristics of the cohort are summarized in Table 1. BMI, age, and menstrual-cycle day of biopsy collection were comparable between the two groups. Fourteen dysregulated miRNAs were identified, of which nine were upregulated and five were downregulated, as presented in Table 2.

### 3.2. Predicted miRNA Target Genes Are Enriched in Biological Pathways Related to Cell Migration and Proliferation

**Figure 2:**
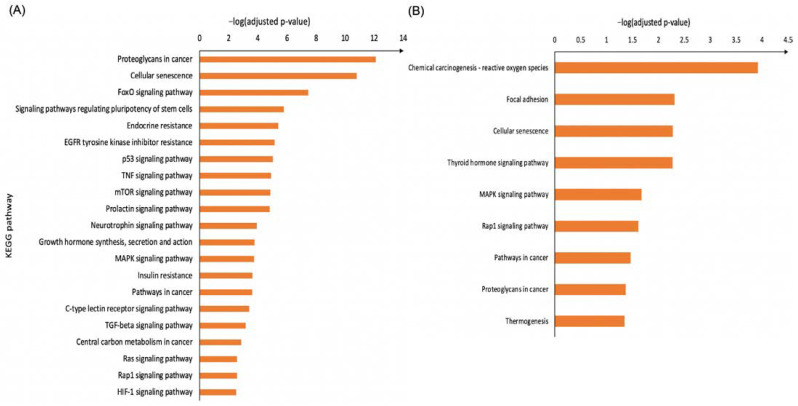
Selected KEGG signaling pathways significantly enriched for the upregulated (**A**) and downregulated (**B**) miRNA’s target genes. The pathways presented have been selected based on involvement in endometriosis, according to the published literature. In Figure 2A, the pathways have been further sorted based on involvement in cell migration and proliferation. A *p*-value of 0.05 was considered statistically significant.

To explore the biological functions of the dysregulated endometrial miRNAs in endometriosis, target gene prediction and pathway analysis for up- and downregulated miRNAs were conducted separately, using the web-based tools miRTarBase and g: Profiler. In total, we identified 1431 potential target genes for the upregulated miRNAs and 912 potential target genes for the downregulated miRNAs (Supplementary Table S1). Further, KEGG pathway analysis was performed to identify potential signaling pathways enriched for the predicted target genes (Supplementary Table S2). For the upregulated miRNA-targeted genes, we identified 54 pathways, of which 21 are strongly related to cell migration and proliferation, as shown in Figure 2A, whereas for the downregulated miRNA-targeted genes, we identified 9out of 19 enriched pathways that have been linked to endometriosis according to the published literature (Figure 2B).

### 3.3. Selection of DE-miRNAs for Ex Vivo and In Vitro Studies

MiR-193b-5p and miR-374b-5p were selected for further examination, based on fold change and consistency in the gene expression profile, and their potential functions reported in the previous literature, with an emphasis on involvement in biological pathways related to migration and proliferation. To begin with, miR-193b-5p presented as the most downregulated endometrial miRNA in women with endometriosis compared to women without endometriosis, with a fold change of −3.7 and a stable gene expression in both groups. This miRNA has also been linked to TGF-beta signaling, and may affect cell proliferation and migration through various biological pathways [21,22]. The most upregulated miRNA in endometriosis endometrium, miR-374b-5p, has previously been shown to regulate cell migration, proliferation, and epithelial-to-mesenchymal transition (EMT) [23,24,25], which are associated with the pathogenesis of endometriosis.

**Figure 3:**
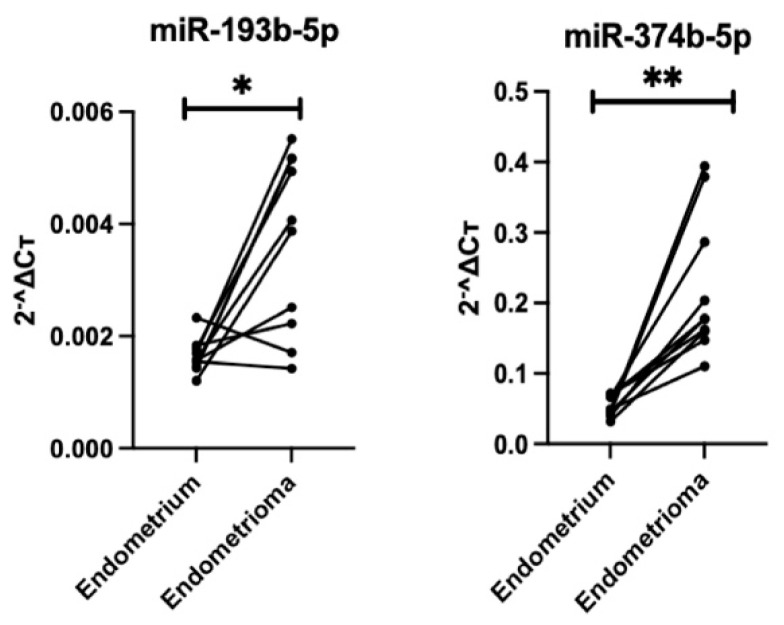
Relative gene expression level of miR-193b-5p and miR-374b-5p in endometrioma (n = 10) compared to paired endometrium (n = 10). Data are presented using a line plot. Wilcoxon matched-pairs signed-rank test was used in the statistical analysis; * *p* < 0.05, ** *p* < 0.01.

The difference in expression levels of the two selected miRNAs between ovarian endometrioma and paired eutopic endometrium was evaluated by qRT-PCR (n = 10; Table 1). As shown in Figure 3, the expression level of miR-193b-5p was significantly upregulated in the endometrioma tissue compared to paired eutopic endometrium (fold change 2.04, * *p* < 0.05). Similarly, the expression level of miR-374b-5p was also significantly upregulated in the endometrioma tissue compared to paired eutopic endometrium (fold change 4.00, ** *p* < 0.01).

### 3.4. Overexpression of miR-193b-5p and miR-374b-5p Does Not Affect the Proliferation of 12Z and HESC Cells

It has been suggested that abnormal proliferation of ectopic endometrial cells may contribute to endometriosis [26]. We therefore wanted to explore whether overexpression of miR-193b-5p and miR-374b-5p would affect endometrial cell proliferation. To test the hypothesis, a BrdU proliferation assay was performed on two endometrial cell lines (12Z and HESC cells) transfected with mimics of the miRNAs. The results indicate that overexpression of miR-193b-5p or miR-374b-5p does not affect the proliferative ability of 12Z and HESC cells compared to the negative control miRNA-transfected cells (Supplementary Figure S1).

### 3.5. Overexpression of miR-193b-5p and miR-374-5p Suppresses Migration of 12Z and HESC Cells

**Figure 4:**
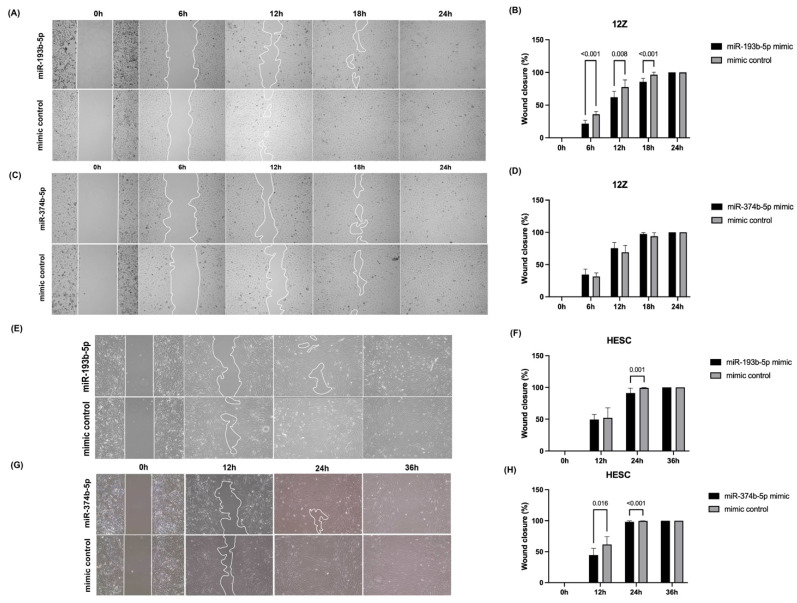
Wound healing assays. (**A**,**C**,**E**,**G**) The representative images of the unclosed wound area at each timepoint. (**B**) In comparison to the mimic control group, the percentage of wound closure was significantly reduced after transfection with miR-193b-5p mimic in 12Z cells. (**D**) No statistical difference in cell migratory ability was observed between miR-374b-5p transfection and control groups in 12Z cells. (**F**) In comparison to the mimic control group, the percentage of wound closure was significantly reduced after transfection with miR-193b-5p mimics in HESC cells at the time point of 24 h. (**H**) In comparison to the mimic control group, the percentage of wound closure was significantly reduced after transfection with miR-374b-5p mimics in HESC cells at time points of 12 h and 24 h. *p* < 0.05 is considered statistically significant. All experiments were repeated three times.

**Figure 5:**
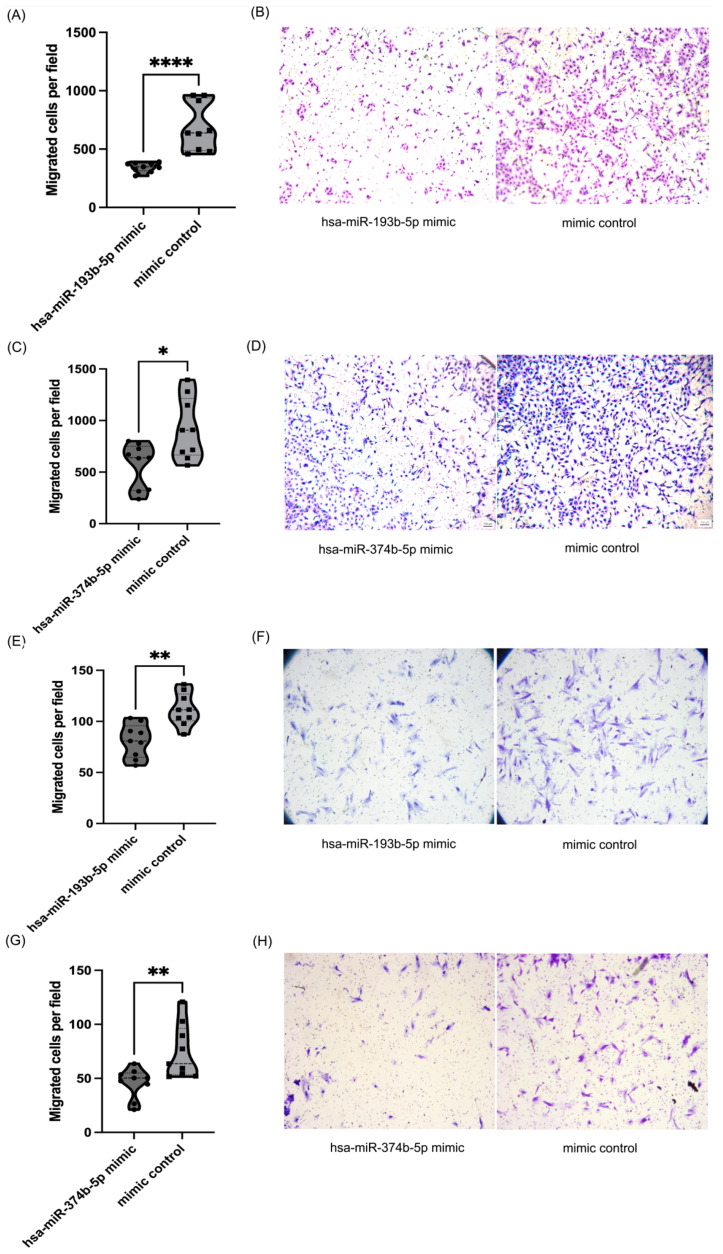
Transwell cell migration assay. (**A**) The migration ability of 12Z cells after hsa-miR-193b-5p mimics transfection compared to the control group. (**C**) The migration ability of 12Z cells after hsa-miR-374b-5p mimics transfection compared to the control group. (**E**) The migration ability of HESC cells after hsa-miR-193b-5p mimics transfection compared to the control group. (**G**) The migration ability of HESC cells after hsa-miR-374b-5p mimics transfection compared to the control group. (**B**,**D**,**F**,**H**) Representative images of the transwell membranes with the migrated cells (5x). n = 9 independent replicates, * *p* < 0.5, ** *p* < 0.01, **** *p* < 0.0001; Mann–Whitney U-test.

Several studies have indicated that aberrant miRNA expression plays an important role in the progression of endometriosis by altering the migration ability of cells [12,27]. Hence, we conducted wound-healing and transwell migration assays to investigate the impact of miR-193b-5p and miR-374b-5p on cell migration of 12Z and HESC cells. As shown in Figure 4, overexpression of miR-193b-5p resulted in a marked decrease in wound closure rate in both 12Z and HESC cells compared to the control group (Figure 4, 12Z *p* < 0.001 at 6 h and 18 h, *p* = 0.008 at 12 h; HESC *p* < 0.001 at 24 h). Overexpression of miR-374b-5p significantly reduced the wound closure rate of HESC cells but not of 12Z cells (Figure 4, HESC *p* = 0.016 at 12 h, *p* < 0.001 at 24 h). To further confirm the above findings, transwell migration assays indicated a significant decrease in the vertical migration capacity of cells following the overexpression of miR-193b-5p and miR-374b-5p in both 12Z and HESC cells (Figure 5).

### 3.6. Identification of Target Genes of miR-193b-5p and miR-374b-5p

To identify the downstream genes targeted by miR-193b-5p and miR-374b-5p, we performed mRNA sequencing on the miRNA-transfected cell lines (HESC and 12Z cells). In the miR-193b-5p transfected 12Z group, 220 upregulated DEGs and 494 downregulated DEGs were identified, and 380 up- and 613 downregulated DEGs were found in the miR-193b-5p HESC group. Meanwhile, 10 up- and 26 downregulated DEGs were found in the miR-374b-5p 12Z group, and 188 up- and 173 downregulated DEGs were found in the miR-374b-5p HESC group (Supplementary Table S3). To understand the biological functions of the identified DEGs, a KEGG pathway enrichment analysis was performed only on the downregulated DEGs, since upregulation of miRNAs will result in suppression of gene expression. The results revealed that 34 signaling pathways were significantly enriched in the miR-193b-5p 12Z group, 84 in the miR-193b-5p HESC group, 4 in the miR-374b-5p 12Z group, and 54 in the miR-374b-5p HESC group (Supplementary Table S4). Enriched signaling pathways relevant to endometriosis include pathways such as the Wnt signaling pathway, regulation of actin cytoskeleton, focal adhesion, and cell adhesion molecules.

In addition, mRNA sequencing was also conducted on the paired endometrioma-endometrium cohort where the two miRNAs had been confirmed to be upregulated. The purpose of the analyses was to confirm whether the miRNA target genes identified in the transfected cell lines were also downregulated in the tissue samples, to increase the robustness of our findings. In total, 6203 DEGs including 3819 up- and 2384 downregulated DEGs were identified (FC >= 2 and FDR < 0.05) (Supplementary Table S3). Pathway analysis showed that 118 KEGG pathways were significantly enriched (Supplementary Table S4). Among the top 15 biological pathways, we identified several pathways highly relevant for cell migration, such as cell adhesion molecules, Rap1 signaling pathways, PI3K-Akt signaling pathway, and focal adhesion (Table 3).

### 3.7. Potential Key miRNA Target Genes Relevant to Cell Migration in Endometriosis

To identify the most likely down-stream target genes of miR-193b-5p and miR-374b-5p responsible for the suppression of cell migration observed in the in vitro experiments, we performed a thorough overlapping analysis among the downregulated DEGs in transfected cells and the downregulated DEGs in the endometrioma cohort. The overlapping genes were further selected based on involvement in cell migration in the published literature. The results of the analyses indicate that the in vitro cell migration is most likely decreased, at least partially, as a result of miR-193b-5p targeting *DKK1*, *MIENI*, *GRB7*, *PIK3R1*, and *KRT19* and by miR-374b-5p targeting *DKK1*, *GRB7*, *PI3K1* and *KRT19* (Table 4).

## 4. Discussion

Our study reveals distinct miRNA expression profiles in the proliferative eutopic endometrium of women with moderate-to-severe endometriosis, highlighting 14 differentially expressed miRNAs. Among these, the most significantly dysregulated microRNAs were miR-374b-5p (upregulated) and miR-193b-5p (downregulated). Further, ex vivo studies showed that miR-374b-5p and miR-193b-5p were both overexpressed in ovarian endometriomas. Functional assays demonstrated that these two miRNAs may have significant regulatory effects on endometrial cell migration, but not proliferation. Furthermore, mRNA sequencing and bioinformatic analysis identified several migration-related genes targeted by these two miRNAs.

To the best of our knowledge, this is the first study applying an unbiased approach using small RNA sequencing to profile microRNA gene expression in the endometrium of women with and without endometriosis. Previous studies have utilized microarrays with a selected panel of miRNA genes, limiting the ability to discover dysregulated miRNA expression in the endometrium of women with endometriosis [28,29,30,31,32]. The dysregulated miRNAs identified in our study through small RNA sequencing partially overlap with those reported in previous microarray-based studies, particularly miR-374b-5p and miR-374c-3p [28,32]. However, the overall concordance between our findings and earlier reports is low [29,30,31]. This discrepancy may result from differences in study design, patient selection (e.g., stage of the disease, menstrual cycle phase), experimental design (e.g., microRNA gene expression profiling method and qRT-PCR endogenous controls), and bioinformatics analyses. These factors underscore the need for standardized approaches in miRNA gene-expression studies related to endometriosis.

Our findings show that miR-193b-5p was downregulated in the eutopic endometrium of women with endometriosis compared to those without, while it was upregulated in endometrioma compared to paired endometrium. This dual regulation suggests a complex role for this miRNA in the pathogenesis of endometriosis. Considering the disparity between ectopic and eutopic endometrium in this condition, we speculate that miR-193b-5p may play different roles, depending on the tissue context. It is likely that the observed upregulation of miR-193b-5p and miR-374b-5p in endometriotic tissue may reflect a physiological response to limit ectopic tissue growth. This notion is supported by our in vitro findings, which showed that overexpression of both microRNAs suppressed endometrial cell migration, and that genes targeted by these miRNAs are enriched in biological pathways critical for cell migration and invasion, such as cell adhesion molecules and TGF-beta signaling. In line with these findings, the previous literature on cancer suggests that these miRNAs may act as tumor suppressors when overexpressed. For instance, the upregulation of miR-193b-5p has been shown to inhibit cell proliferation and migration while promoting apoptosis via the AKT/mTOR pathway [20,21]. Similarly, the upregulation of miR-374b-5p has been suggested to suppress cell migration and invasion and to reverse epithelial–mesenchymal transition [22,23,24]. Additionally, our study highlights several potential key genes likely involved in the pathogenesis of endometriosis by altering cell migration, such as *DKK1*, *GRB7*, *MIEN1*, *PIK3R1*, and *KRT19*. For instance, our findings suggest that upregulation of miR-193b-5p and miR-374b will downregulate *DKK1,* which may contribute to decreased cell migration observed in vitro. This is in line with a recently published study demonstrating that upregulation of *DKK1* promotes cell migration at certain concentrations [33]. The regulation of these genes by miR-193b-5p and miR-374b-5p emphasizes their potential impact on the migratory behavior of endometrial cells.

From a genetic perspective, one review paper summarized the genetic contributions to endometriosis and highlighted several genetic variants associated with endometriosis, shedding light on molecular pathways and mechanisms involved [34]. Some of the highlighted genetic variants were *WNT4* and *FN1*, which have been implicated in endometrial tissue remodeling and cell migration. These processes are central to endometriosis and are also highlighted in this study. Our data also revealed that *FN1* was significantly upregulated in endometrioma compared to paired endometrium (Table S3). However, further research is required to understand the role of these genetic and epigenetic variants involved in endometriosis pathogenesis.

Our study has several strengths. We used an unbiased screening approach to identify differentially expressed miRNAs in the endometrium of women with and without endometriosis and further studied selected miRNAs ex vivo in paired endometriomas and eutopic endometrium, as well as functionally in vitro to increase the robustness of our findings. To reduce sample heterogeneity, we focused on samples from the proliferative phase of the menstrual cycle, and restricted our inclusion criteria to cases of moderate-to-severe endometriosis (stage III–IV). Additionally, all participants were laparoscopically confirmed for the presence or absence of endometriosis. We utilized paired samples to control for intra-sample variability. These meticulous selection criteria enhance the reliability and minimize the variability of our study cohorts. However, some limitations should be acknowledged. The sample size was relatively small, and the functional experiments were performed in cell lines. To partially overcome this limitation, we included two endometrial cell lines to cover both the major cell types of the endometrium, e.g., stromal and epithelial cells. Future functional studies should include primary cells from patients with endometriosis to increase the reliability of the in vitro findings.

In summary, with a robust understanding of the dysregulated miRNAs within the eutopic endometrium of patients with endometriosis, our study highlights the pivotal role of miR-193b-5p and miR-374b-5p in regulating cell migration in endometriosis. Further research is needed to elucidate the complex miRNA-gene regulatory networks. Although the therapeutic potential of miRNAs has not yet been evaluated in endometriosis, our findings lay the foundation for future development of miRNA-based drugs to halt or even reverse this condition.

## 5. Conclusions

We report for the first time the detection of several dysregulated miRNAs in proliferative eutopic endometrium using an unbiased screening approach. By investigating the functional roles of identified differentially expressed miRNAs, notably miR-193b-5p and miR-374b-5p in vitro, we found that these two miRNAs play significant suppressor roles on cell migration ability, and we did not observe changes in cell proliferation in endometrial cell lines. Through comprehensive bioinformatic analysis, we highlighted several biological pathways and their potential involvement in the pathogenesis of endometriosis via regulating key genes. These findings provide unique insights into the molecular basis of endometriosis.

## Figures and Tables

**Table 1:** Clinical characteristics of the two cohorts.

	Cohort 1		Cohort 2
	Endometriosis (n = 15)	Non-Endometriosis Controls (n = 17)	Endometrioma (n = 10)
Age (mean, range), years	33 (24–43)	33 (25–38)	36 (27–42)
BMI (mean, SD ^1^), kg/m^2^	22 ± 3.4	24 ± 4.7	21 ± 2.8
Smoker	1 (6.7%)	3 (17.6%)	2 (20.0%)
Menstrual cycle day ^2^, mean,(range), days	8.2 (2–12)	8.8 (1–13)	9.9 (4–12)
Infertility rate	7 (50%)	0	3 (30%)
Endometriosis stage			
- III	9 (60%)	na	7 (70%)
- IV	6 (40%)	na	3 (30%)

^1^ SD: standard deviation; ^2^ menstrual cycle day: the menstrual cycle day when endometrial biopsies were collected; na: not applicable.

**Table 2:** Differentially expressed miRNAs in eutopic endometrium of women with endometriosis compared to women without endometriosis.

	miRNA ID	Fold Change	Adjusted *p*-Value
Upregulated miRNAs	hsa-miR-374b-5p	3.9	0.012
	hsa-miR-1307-5p	3.7	0.003
	hsa-miR-561-5p	3.6	<0.000
	hsa-miR-144-3p	2.8	0.015
	hsa-miR-19a-3p	2.7	0.015
	hsa-miR-18b-5p	2.6	0.012
	hsa-miR-136-5p	2.3	0.018
	hsa-miR-96-5p	2.2	0.020
	hsa-miR-101-3p	2.0	0.025
Downregulated miRNAs	hsa-miR-193b-5p	−3.7	<0.000
	hsa-miR-374c-3p	−3.6	0.006
	hsa-miR-320b	−2.5	0.005
	hsa-miR-320a-3p	−2.2	<0.000
	has-miR-4521	−2.2	0.020

**Table 3:** Top 15 biological pathways significantly enriched for the differentially expressed mRNA genes identified through mRNA sequencing of the paired endometrioma–endometrium cohort.

Gene Set	Description	Enrichment Score	*p*-Value
Path:hsa04380	Osteoclast differentiation	17.165	3.51× 10^−8^
path:hsa04145	Phagosome	17.119	3.67 × 10^−8^
path:hsa04514	Cell adhesion molecules	16.817	4.97 × 10^−8^
path:hsa05150	Staphylococcus aureus infection	15.827	1.34 × 10^−7^
path:hsa05166	Human T-cell leukemia virus 1 infection	13.484	1.39 × 10^−6^
path:hsa04924	Renin secretion	12.517	3.66 × 10^−6^
path:hsa04151	PI3K-Akt signaling pathway	12.237	4.84 × 10^−6^
path:hsa04640	Hematopoietic cell lineage	12.216	4.95 × 10^−6^
path:hsa04015	Rap1 signaling pathway	10.953	1.75 × 10^−5^
path:hsa04662	B cell receptor signaling pathway	10.922	1.81 × 10^−5^
path:hsa04512	ECM-receptor interaction	10.900	1.83 × 10^−5^
path:hsa05323	Rheumatoid arthritis	10.279	3.43 × 10^−5^
path:hsa04713	Circadian entrainment	10.020	4.42 × 10^−5^
path:hsa04510	Focal adhesion	9.901	5.01 × 10^−5^
path:hsa05200	Pathways in cancer	9.833	5.36 × 10^−5^

**Table 4:** Selected overlapped DEGs relevant to cell migration.

Gene Symbol	Gene Name	Expression Direction
*DKK1*	Dickkopf WNT Signaling Pathway Inhibitor 1	Downregulated in miR-193b-5p transfected 12Z Downregulated in miR-374b-5p transfected 12Z Downregulated in miR-193b-5p transfected HESC Downregulated in miR-374b-5p transfected HESC Downregulated in endometrioma tissue
*MIEN1*	Migration And Invasion Enhancer 1	Downregulated in endometrioma tissue Downregulated in miR-193b-5p transfected 12Z
*GRB7*	Growth Factor Receptor-Bound Protein 7	Downregulated in miR-193b-5p transfected 12Z Downregulated in endometrioma tissue
*PIK3R1*	Phosphoinositide-3-Kinase Regulatory Subunit 1	Downregulated in miR-193b-5p transfected HESC Downregulated in miR-374b-5p transfected HESC Downregulated in endometrioma tissue
*KRT19*	Keratin 19	Downregulated in miR-193b-5p transfected 12Z Downregulated in miR-374b-5p transfected HESC Downregulated in endometrioma tissue

## Data Availability

Sequence data have been deposited in the NCBI’s Gene Expression Omnibus and are accessible through the following GEO Series accession numbers: GSE275002 for small RNA sequencing data and GSE279835 for the mRNA sequencing data.

## Supplementary Materials

The following supporting information can be downloaded at: https://www.mdpi.com/article/10.3390/biom14111400/s1, Figure S1: BrdU proliferation assay result; Table S1: DE-miRNAs predicted target gene list; Table S2: Target gene KEGG pathway analysis list; Table S3: mRNA-seq DEGs; Table S4: mRNA-seq DEG KEGG.
