# Supplementary material for: miR-193b-5p and miR-374b-5p Are Aberrantly Expressed in Endometriosis and Suppress Endometrial Cell Migration In Vitro
Source: Biomolecules. 2024 Nov 3;14(11):1400. doi: 10.3390/biom14111400 (PMC11592355; doi:10.3390/biom14111400)

Supplementary Figure S1. BrdU proliferation assay. Incorporation of BrdU (OD450) in 12Z and HESC cells as measured by ELISA after miR-193b-5p or miR-374b-5p mimic transfection for 48 hours. Violin plot. n = 9 independent replicates; ns, not significant; Mann-Whitney U-test.

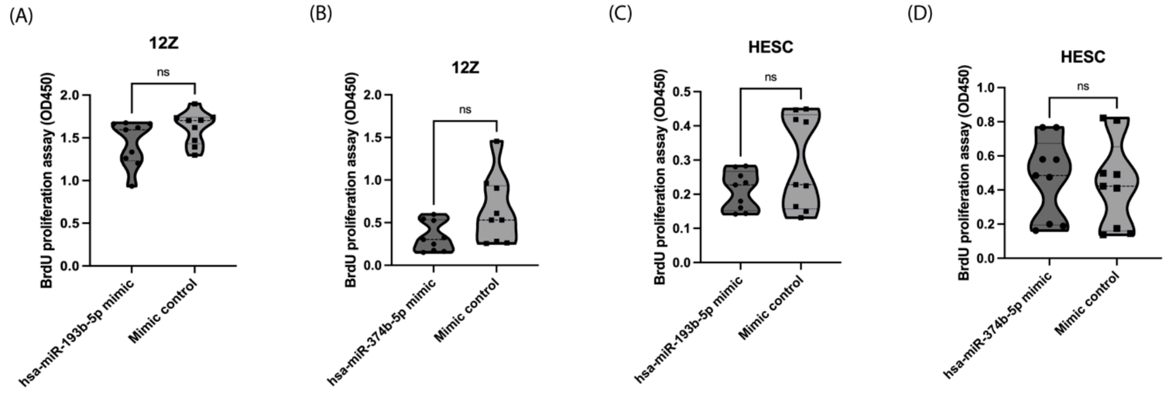

Supplement: Supplementary file 1 [file biomolecules-14-01400-s001.zip › biomolecules-3238183-supplementary/Supplementray/Supplementary Figure S1_BrdU proliferation assay results.pdf]
